# On Diseases of the Liver

**Published:** 1853

**Authors:** 


Art. XI.—*On Diseases of the Liver.* By George Budd, M. D., F. R. S. Philadelphia: Blanchard & Lea. 1853.

The first edition of this work, published some years ago in this country, was received by the press and the profession with great favor, and we are glad that a second has rendered its valuable store of information accessible to those who are not in possession of the volume. Diseases of the Liver, once so prolific a subject, have been of late years very much neglected by medical writers, and such a treatise as the monograph before us was greatly needed. Our knowledge both of the structure and diseases of this important organ was exceedingly vague when most of the former treatises were written, and the current pathology of a period just past, which ascribed most of the diseases of the abdominal viscera, as well as all fevers, to disorder of the liver, demanded new and more accurate investigation. The microscope has thrown a flood of light upon the minute structure of this organ; its study in the laboratory and in the lower series of the animal kingdom has increased our knowledge of its function, so that the writer of the present day enjoys great advantages over his predecessors.

Dr. Budd begins with an account of the intimate structure of the liver, as revealed by the researches of Kiernan, Bowman, Henle, and Muller, which forms an appropriate introduction to the study of its diseases. Congestion is the first morbid condition of the liver treated of. The writer remarks:—

"Congestion of the liver from mechanical impediment to the onward current of the blood is generally brought under our notice, not as a disease of itself, but as a consequence and a complication of valvular disease of the heart, or of some other condition that prevents the free passage of the blood through the chest. But, although a secondary disorder, its results are very important. If it continue long, it leads to bilious contamination of the blood, often already impure by defective action of the lungs and the kidneys; and in other ways much aggravates the condition of the patient.

The congestion of the liver may be relieved directly by general or local bleeding, or by medicines, such as sulphate of magnesia and bitartrate of potash, which cause a copious drain from the portal system of veins; and, indirectly, by medicines, such as small doses of blue pill, which increase the secretion of bile. In cases of dropsy from disease of the heart, when the liver is gorged and the complexion sallow, small doses of blue pill, in conjunction with diuretics or purgatives, are often productive of extraordinary benefit.

Under such circumstances, it is almost needless to remark, it is very important that the patient should take very sparingly of fermented drinks, and abstain from rich dishes, and indeed, from all articles of food likely to add to the congestion of the liver.

Hitherto we have considered only that kind of congestion which results from mechanical hindrance to the backward current of the venous blood. But congestion—that is, undue accumulation of blood in the vessels—may result from totally different conditions.

The large vessels serve merely as channels to convey to the different tissues of the body the blood from which the materials of their nutrition are drawn. The process of nutrition is dependent on a mutual affinity between the blood and the tissues, by virtue of which each part withdraws from the blood through the thin walls of the capillary vessels those materials which its proper nutrition requires. And the equable distribution of the blood through the body depends not merely on its more obvious conditions—

on the propulsive power of the heart, on the suction power of respiration, and on there being a free passage for it through the arteries and the veins; but also on this mutual action, or affinity, between the blood and the tissues, which is being constantly exerted in every part of the body as long as its nutrition continues. Modifications of this affinity, leading to congestion, or undue fulness of the vessels, may result from changes either in the tissues or in the blood. Thus, if a part be injured in any way—if the skin be cut, or a bone be broken—provided the vitality of the tissues be not destroyed, there is immediately set up a process of inflammation, or of repair, and one of the first results of this process is an increased flow of blood to the part and a turgid state of its vessels. So, again, if any part be the seat of a cancer or of any other morbid growth, there is at once, by virtue of this increased and faulty nutrition of the tissues, an increased flow of blood to the part, and, after a time, the vessels of that part are found to have grown larger. So, indeed, it is generally: wherever an important vital process is going on, there is an increased flow of blood to the part, and a congestion, if it may be so termed, or an accumulation of blood in the vessels, by which the vital action is maintained. And there can be no doubt that this increased flow of blood, and this turgescence of the vessels, is secondary to the vital action, and the result of it; and that it is caused by the modification of the affinity between the blood and tissues which the action in question produces."

Hemorrhage has been generally regarded as a common result of affections of the liver. Dr. Budd thinks that it is exceedingly rare:—

"Now and then, however, it does occur; more especially, according to the observations of Rokitansky, in children, when the liver is much congested from suffocative catarrh, or some other condition that impedes the circulation through the chest. The hemorrhage may take place in the substance of the liver or at its surface, or in both situations at once. When it takes place in the substance of the liver, it may cause but little pain, and do but little other mischief. After a time, the blood, like blood effused elsewhere, is absorbed, and no sign of the accident remains; or the only sign of it that remains is a scar, the origin of which can seldom be traced. The hemorrhage thus escapes detection. When hemorrhage takes place at the surface of the liver, the blood may collect under the capsule, and form a palpable tumor; or may even rupture the investing membranes, and thus become effused into the peritoneal sac. It then, of necessity, causes much pain and tenderness, which are more or less widely diffused, according to the nature of the injury. The pain and tenderness are, however, seldom of long continuance. The effused blood does not inflame the serous membrane, and, in consequence, the pain and tenderness disappear entirely, or much abate, in the course of a few days."

The chapter on inflammation of the liver is a very elaborate and important one. Inflammation of the liver may be excited by a great variety of causes, and assume a variety of forms. Abscess may be the result, and this is found frequently to ensue upon surgical operations. Upon this subject Dr. Budd remarks:—

"It may be considered then established, that the abscesses which form in the liver and other organs, after surgical operations and injuries of the head or limbs, are owing to suppurative inflammation of a vein, and the consequent contamination of the blood by pus. The globules of pus, mingled with the blood, are conveyed to the capillary vessels of the lungs, and, it would seem, by becoming mechanically arrested there, excite each circumscribed inflammation and abscess. If any of the globules pass through the capillaries of the lungs to the left side of the heart, they are sent in the arterial current to other organs, and, becoming arrested in the capillaries of these organs, excite, as in the lungs, inflammation of limited extent, rapidly passing on to abscess.

These scattered abscesses are most commonly found after operations or injuries, because suppurative inflammation of the inner surface of a vein is most commonly caused by mechanical injury of its coats; but they may obviously result from suppurative phlebitis set up in any other way. I have met with two instances in which scattered abscesses in various organs seemed to result from a collection of pus that had formed; from some cause which I could not discover, between the periosteum and bone of the upper arm; another instance, in which their source was probably a large tuberculous cavity in the lungs.

Perhaps then, we are justified in concluding, in all cases in which we find collections of pus rapidly formed in different parts of the body, that the immediate cause of these scattered inflammations is some irritating substance conveyed there by the blood; and in most of the cases where the abscesses in the lungs are small and circumscribed, that this irritating substance is pus, derived from inflammation of the inner surface of a vein.

In cases in which we cannot find the inflamed vein, the facts that the abscesses are scattered in the same way, and occupy the very same anatomical seat as in those cases in which the source of the pus is known—that this kind of dissemination and the anatomical seat occupied are also the same as in the case of injected mercury and secondary cancer — are conclusive in showing that the agent arrives by the blood, and almost conclusive that this agent is a pus-globule.

The proportion of cases of this kind, in a given number of cases of abscess of the liver, will, of course, vary with the frequency of abscess of the liver from other causes.

In India, where other powerful causes of abscess of the liver are in operation, the proportion will be small. In the cases published by Annesley, there is not one that we can, from his description, place in this category.

In the seventeen cases that have fallen under my own care at the Dreadnought, and in King's College Hospital, there is only one that clearly belongs to this head. In that instance, abscess of the liver, with abscesses of the lungs and collections of pus in various joints, resulted from phlebitis caused by the operation of bleeding.

In the sixteen cases collected by Louis and Andral, in Paris, where abscess of the liver from other causes is less frequent, there are four which may be placed in this category;—one in which the abscesses were consequent on venesection (Louis, Obs. 2); another in which they were consequent on childbirth (Louis, Obs. 1); a third (Andral, Obs. 23), where, with abscesses of the liver, there was lobular pneumonia of the left lung, gray hepatization of the right, and pus between the vertebral column and pharynx; a fourth (Andral, Obs. 26), in which there was gray hepatization of the lower lobe of the left lung, and pus in the mediastinum."

We have no hesitation in expressing the opinion, that this work of Dr. Budd's is one of the most trustworthy monographs ever presented to the profession, and that it ought to be in the library of every physician.

